# Single‐Atom Sites on MXenes for Energy Conversion and Storage

**DOI:** 10.1002/smsc.202100017

**Published:** 2021-05-07

**Authors:** Yanglansen Cui, Zhenjiang Cao, Yongzheng Zhang, Hao Chen, Jianan Gu, Zhiguo Du, Yongzheng Shi, Bin Li, Shubin Yang

**Affiliations:** ^1^ School of Materials Science and Engineering Beihang University 100191 Beijing China

**Keywords:** catalysis, cation defects, covalent bonds, MXenes, rechargeable batteries, single atoms

## Abstract

Single‐atom sites on MXenes (SASs‐MXenes) have attracted widespread attention for energy storage and conversion due to their highest atom utilization efficiency, intriguing intrinsic properties, unusual performance, and improved robustness. In addition, the large surface area and abundant anchor sites make MXenes ideal substrates for supporting single atoms via covalent interaction. Herein, the main strategies for synthesis of SASs‐MXenes are first summarized, which cover capturing single atoms by cation vacancies, coordinating single atoms with heterodopants, and inheriting single atoms from MAX phases. Then, disclosing the crucial roles SASs‐MXenes play in tuning the kinetics and thermodynamics of various catalytic reactions, i.e., hydrogen evolution reaction, nitrogen reduction reaction, CO_2_ reduction reaction, CO_2_ functionalization, polysulfide conversion, and other redox reactions involved in rechargeable batteries, is focused on. Finally, the challenges and future opportunities for developing highly active SASs‐MXenes are discussed.

## Graphical abstract

Single‐atom sites on MXenes (SASs‐MXenes) show great potentials in various energy conversion and storage applications due to their unique electronic structures and tunable coordination environments. Herein, the synthetic strategies for SASs‐MXenes are summarized and their essential roles in these energy‐related reactions are revealed. Finally, the challenges and prospects to develop high‐performance SASs‐MXenes are presented.

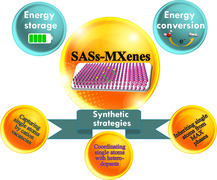

## 1 Introduction

With growing demand for utilizing the clean and renewable energy for practical applications in our daily life, it is motivating to develop the highly efficient energy‐storage and conversion systems.^[^ ^1^ ^]^ Recently, single‐atom sites (SASs) anchored on 2D materials exhibit great potential in energy‐related applications due to their highly exposed active centers and maximized atom utilization.^[^ ^2^ ^]^ Also, the atomically dispersed active sites afford an ideal platform to directly relate their structural characteristics to the performances at atomic level.^[^ ^3^ ^]^ However, the single atoms tend to aggregate into nanoclusters or particles during preparation due to their high surface energies.^[^ ^4^ ^]^ Thus, the immobilization of the SASs on 2D substrates via strong bonding is the prerequisite for their utilization in various applications.^[^ ^5^ ^]^ Currently, various 2D substrates have been adopted to support the single atoms, such as graphene, MXenes, transition metal dichalcogenides (TMDs), and layered double hydroxides (LDHs).^[^ ^6^ ^]^ Among them, MXenes possess many distinct advantages. 1) The naturally formed atomic cation defects and adjustable terminals provide abundant spots for capturing and stabilizing single atoms. 2) The incorporation of single atoms redistributes the local electronic structures, leading to favorable electronic properties. 3) The strong covalent interaction between single atoms and MXenes induces the unusual performance.^[^ ^7^ ^]^

To date, a series of single heteroatoms have been incorporated on MXenes, such as Co, Cu, Ru, and Pt, which perform decently when used as catalysts for hydrogen evolution reaction (HER), nitrogen reduction reaction (NRR), CO_2_ functionalization, and CO_2_ reduction reactions (CRR).^[^ ^8^ ^]^ In addition, the SASs‐MXenes have been involved in the redox reactions for different energy‐storage devices, such as lithium metal batteries (LMBs), lithium‐sulfur (Li‐S) batteries, sodium‐ion batteries (SIBs), and zinc‐ion batteries (ZIBs).^[^ ^9^ ^]^ In this Review, we critically review the current progress on SASs‐MXenes as highly active centers toward a range of reactions. First, the synthetic strategies for SASs‐MXenes are summarized, which can be categorized into three main types, including capturing single atoms by cation vacancies, coordinating single atoms with heterodopants, and inheriting single atoms from MAX phase. Then, we mainly reveal the exact role SASs‐MXenes play in regulating the kinetics and thermodynamics of different reactions, offering a deep understanding from mechanism aspects. Finally, we discuss the existing bottlenecks for controllable synthesis of SASs‐MXenes, and the feasible opportunities for the future development of SASs‐MXenes are highlighted.

## 2 Synthetic Strategies for SASs‐MXenes

As the isolated single atoms show high mobility due to their high surface energy, it is crucial to avoid the formation of nanoclusters or nanoparticles during preparation of SASs‐MXenes.^[^ ^10^ ^]^ The cation vacancies generated on MXenes during the etching process can serve as ideal points for capturing the single atoms.^[^ ^11^ ^]^ Also, the oxygen‐rich surface functional groups on MXenes are expected to adsorb the cations for the following stabilization of single atoms. Moreover, the different metal sources with tunable ratios can be added during the synthesis of MAX phase. After selectively etching MAX phase, the SASs on MXenes are maintained and exposed. Benefiting from the structural features of MXenes and diversified compositions of MAX phases, the following synthetic strategies are developed.

### 2.1 Capturing Single Atoms by Cation Vacancies

Since Zhang et al. first reported the concept of SASs and fabricated the isolated Pt single atom onto the defects of FeO_*x*_, the defective sites have been widely used to capture single atoms.^[^ ^12^ ^]^ It has been proved that the cation defects generally exist in MXenes, which are formed during the harsh etching process of “A” layers from MAX phase.^[^ ^13^ ^]^ Chen and coworkers found that the Ti vacancies in Ti_3−*x*_C_2_T_*y*_ nanosheets possess a high reducing ability, which can stabilize the single atom through a self‐reduction process, resulting in a Pt loading of ≈0.2 wt%. In addition, a series of single atoms (Pt, Ru, Ir, Rh, and Pd) on MXenes can be synthesized by this method at room temperature.^[^ ^14^ ^]^

**Figure 1:**
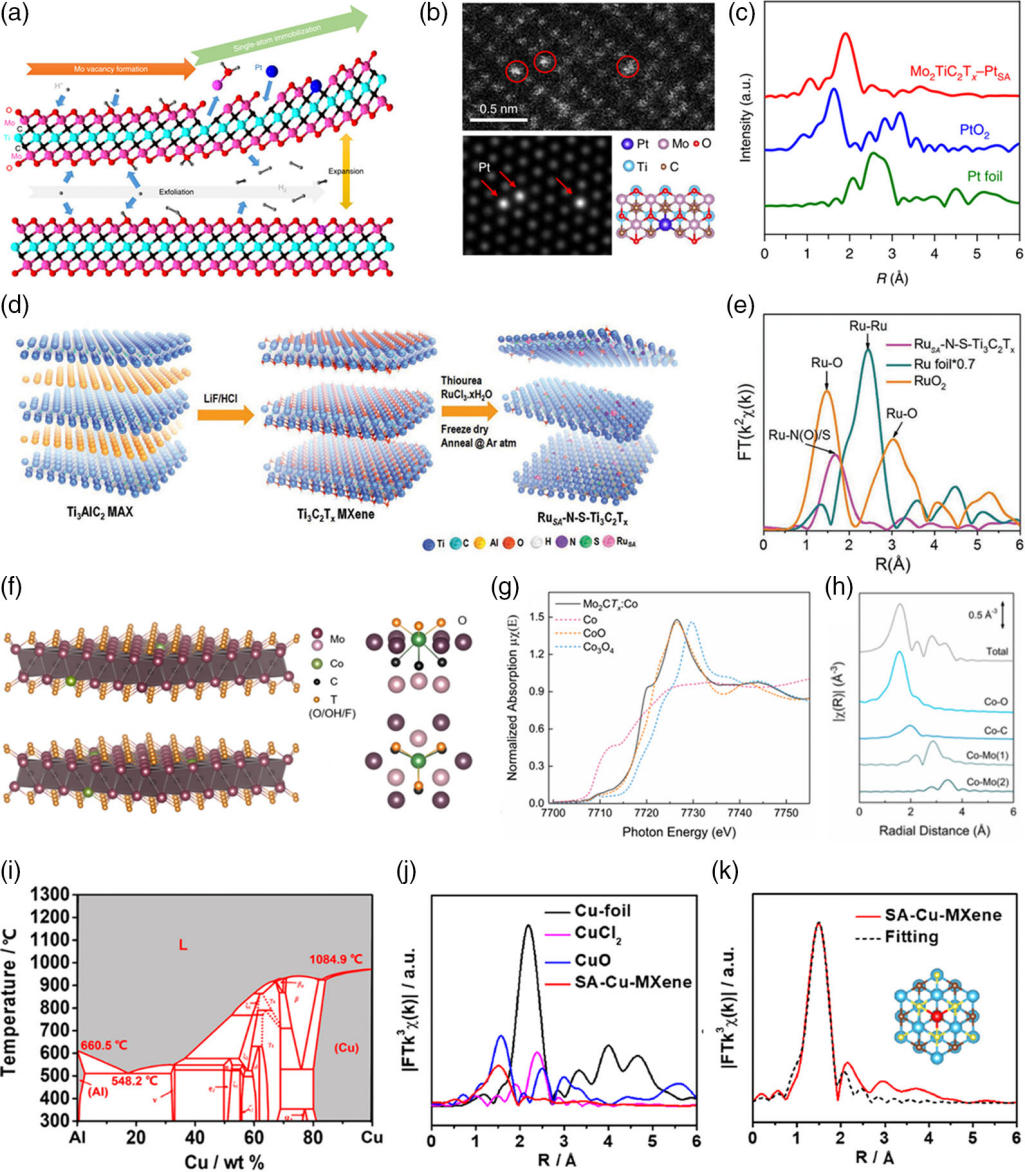
Synthesis strategies for SASs‐MXenes. a) Scheme of the synthesis procedures for Mo_2_TiC_2_O_2_−Pt_SA_. b) HAADF–STEM image of Mo_2_TiC_2_T_*x*_−Pt_SA_ at high magnification. c) Comparison of the FT‐EXAFS spectra of Pt foil, PtO_2_, and Mo_2_TiC_2_T_*x*_–Pt_SA_. a–c) Reproduced with permission.^[^ ^15^ ^]^ Copyright 2018, Springer Nature. d) Scheme of synthetic route for Ru_SA_−N−S−Ti_3_C_2_T_*x*_ catalyst. e) A comparison of the FT‐EXAFS spectra of Ru foil RuO_2_ and RuSA−N−S−Ti_3_C_2_T_*x*_. d,e) Reproduced with permission.^[^ ^20^ ^]^ Copyright 2019, Wiley‐VCH. f) Atomic structure of Mo_2_CT_*x*_:Co (left) and two projections of the coordination environment of cobalt in Mo_2_CT_*x*_:Co (right). g) Comparison of the XANES spectra of Mo_2_CT_*x*_:Co, Co, CoO, and Co_3_O_4_ at Co *K*‐edge. h) The scattering paths used for EXAFS fitting. f–h) Reproduced with permission.^[^ ^23^ ^]^ Copyright 2019, American Chemical Society. i) Al−Cu binary phase diagram. j) *k*3‐weighted Fourier transform of the EXAFS spectra of SA‐Cu‐MXene compared with Cu foil, CuO, and CuCl_2_ references. k) EXAFS fitting curves of SA‐Cu‐MXene. i–k) Reproduced with permission.^[^ ^24^ ^]^ Copyright 2021, American Chemical Society.

In another work, Wang and coworkers reported an electrochemical method to in situ deposit the single Pt atoms on the Mo vacancies, producing single platinum atoms immobilized on Mo_2_TiC_2_T_*x*_ nanosheets (Mo_2_TiC_2_T_*x*_−Pt_SA_).^[^ ^15^ ^]^ A typical three‐electrode system was used, in which a Pt foil and Mo_2_TiC_2_T_*x*_ on carbon paper (CP) worked as counter electrode and working electrode, respectively. As shown in **Figure** **1**a, the Mo vacancies are generated during the electrochemical exfoliation process and Pt single atoms dissolved from the counter electrode are captured by these Mo vacancies at the same time, which is driven by the change of surface chemical composition of MXenes altered by hydrogen cation (H^+^) in acid. The high‐angle‐annular dark‐field–scanning transmission electron microscopy (HAADF–STEM) image at high magnification (see Figure 1b) shows that the isolated Pt atoms (bright spots) exactly occupy on Mo positions. The extended X‐ray absorption fine structure (EXAFS) shows the prominent shell at 1.9 Å, which corresponds to the Pt—C bond (Figure 1c). The fitting results also suggest that the Pt atom is seated on the Mo vacancies and bonds with three adjacent C atoms. Benefiting from the large amounts of Mo vacancies on the surface of MXenes nanosheets, a higher mass loading of Pt single atoms (1.2 wt%) can be achieved, in comparison with that (≈0.2 wt%) prepared by the previous self‐reduction method. The quantity of vacancies directly determines the theoretical loading of single atoms on MXenes. Creating abundant vacancies is key to achieve SASs‐MXenes with high metal loading.

### 2.2 Coordinating Single Atoms with Heterodopants

Except the cation defects, the surface‐terminated functional groups can be also used to anchor the single atoms via the strong coordination interaction. MXenes are generally synthesized by removing the “A” element from the MAX phase.^[^ ^16^ ^]^ During the etching process of “A” layers, the “M” layers will be exposed and terminated with functional groups.^[^ ^17^ ^]^ These functional groups have a great impact on the intrinsic properties of MXenes.^[^ ^18^ ^]^ Moreover, these terminated groups on MXenes can be exchanged, enabling the adsorption of various cations, which can be further stabilized by heterodopants.^[^ ^19^ ^]^ Jr‐Hau He and coworkers reported a synthesis method by taking advantages of the coordination interaction between single atoms and MXenes.^[^ ^20^ ^]^ The scheme of synthesis procedure is shown in Figure 1d. First, the MXene (Ti_3_C_2_T_*x*_) was prepared via selectively etching the Al layers from Ti_3_AlC_2_ MAX phase by LiF/HCl mixture. Then, Ti_3_C_2_T_*x*_ was mixed with RuCl_3_·*x*H_2_O and thiourea. After freeze drying the mixture, a foam was obtained and annealed at 500 °C under Ar atmosphere, obtaining the atomically dispersed ruthenium single atoms (Ru_SA_) on the Ti_3_C_2_T_*x*_ support. The Fourier‐transform EXAFS FT‐EXAFS spectrum (Figure 1e) revealed that Ru_SA_ is coordinated with both N and S species on the Ti_3_C_2_T_*x*_ MXene (Ru_SA_‐N‐S‐Ti_3_C_2_T_*x*_). In another study, the atomic Sn^4+^ was anchored on MXene (V_2_C) via the V—O—Sn bond.^[^ ^21^ ^]^ The decoration of Sn^4+^ leads to a highly improved performance as electrode material for lithium‐ion batteries (LIBs), possessing a capacity of 1284.6 mAh g^−1^ at 0.1 A g^−1^. The intrinsic electronic properties of SASs closely depend on their coordination environments, which has great impact on their catalytic performances.^[^ ^22^ ^]^ This strategy provides opportunities to regulate the dopants to achieve desirable catalytic performances.

### 2.3 Inheriting Single Atoms from MAX Phase

Unlike the previous bottom‐up strategies, Müller and coworkers developed a top−down method to prepare cobalt‐substituted MXene (Mo_2_CT_*x*_:Co), in which the single atoms were introduced during the preparation of MAX phase.^[^ ^23^ ^]^ First, the bimetallic β‐Mo_2_C:Co is prepared via carburizing a mixed hydrate consisting of (NH_4_)_6_Mo_7_O_24_·4H_2_O and Co(NO_3_)_2_·6H_2_O under H_2_/CH_4_ (volume ratio of 8:2) atmosphere at 750 °C for 3 h and then followed by a two‐step method involving the synthesis of Mo_2_Ga_2_C:Co MAX phase and hydrofluoric acid (HF) etching process. Figure 1f shows the schematic atomic structure of Mo_2_CT_*x*_:Co. This method provides a controllable route to prepare the mid‐to‐late transition metal (TM)‐substituted 2D carbides (MXenes). The average oxidation state of cobalt in Mo_2_CT_*x*_:Co was indicated by the X‐ray absorption near‐edge structure (XANES) spectrum at Co *K*‐edge (Figure 1g), showing a valence state of Co^2+^. Moreover, the EXAFS spectrum combined with fitting results of the first shell for Mo_2_CT_*x*_:Co at Co *K*‐edge indicates that Co is coordinated with both O and C, confirming the existence of isolated Co center (Figure 1h).

In addition, our group preconfined the active atoms into A layers with specific ratios during preparation of MAX phase.^[^ ^24^ ^]^ As shown in Figure 1i, Cu and Al can form a liquid molten alloy with a tunable molar ratio above 1100 °C, diffusing into the single‐atom‐thick A layers of MAX phase. After removing the Al element in MAX phase, the Cu atoms are maintained on MXenes (SA‐Cu‐MXene). The Fourier‐transformed EXAFS spectra and corresponding fitting results (Figures 1j,k) indicate that the isolated Cu atom is coordinated with three O atoms. Moreover, we found that the Cu particles can be formed with increased Cu content (Al/Cu molar ratio of 9:1) in MAX phase, resulting in the decreased catalytic performance.

As the preparation of MAX phase always requires a high energy input, various metal sources can be rationally added based on their phase diagrams. After annealing and HF etching, the single atoms will be retained in MXenes. Note that the ratio of different metal sources is critical to prevent the formation of clusters or particles.

## 3 SASs‐MXenes for Energy Conversion

SASs‐MXenes have been widely studied as catalysts for a variety of conversion reactions.^[^ ^25^ ^]^ The incorporation of SASs not only directly introduces highly active centers but also brings the redistribution of the local electronic structure of MXenes, exhibiting decent catalytic performance.^[^ ^26^ ^]^ The strong interaction between the MXenes and SAS can induce unique catalytic activity and selectivity.^[^ ^27^ ^]^ In this section, we critically review the advances in SASs‐MXenes for catalyzing several key reactions, including HER, NRR, CRR, and functionalization of CO_2_. We mainly focus on disclosing how this strong interaction between single atoms and MXenes can synergistically determine the high catalytic performance.

### 3.1 Optimizing the Adsorption Energy of Key Intermediates by SASs‐MXenes

The catalytic reactions are always accompanied with adsorption and desorption of reaction intermediates.^[^ ^28^ ^]^ For HER, the Gibbs free energy for hydrogen adsorption (Δ*G*_H*_) is a widely accepted descriptor to evaluate the catalysts. The optimal value of |Δ*G*_H*_| is 0, which benefits both H* adsorption and desorption. Pt‐based materials are the state‐of‐the‐art catalysts for HER with a small |Δ*G*_H*_|. Interestingly, the single‐atom catalysts often deliver unusual catalytic behavior compared with its bulk form.

**Figure 2:**
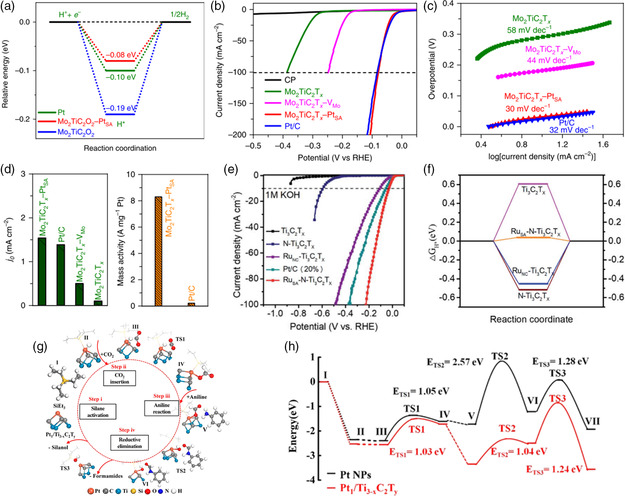
The adsorption energy of key intermediates on SASs‐MXenes and corresponding catalytic performances. a) Comparison of calculated Δ*G*_H*_ for Mo_2_TiC_2_O_2_, Mo_2_TiC_2_O_2_−Pt_SA_, and Pt/C. Comparison of b) polarization curves and c) Tafel slopes of CP, Mo_2_TiC_2_T_*x*_, Mo_2_TiC_2_T_x_−V_Mo_, Mo_2_TiC_2_T_*x*_−Pt_SA_, and Pt/C (40%). d) Comparison of mass activity of Pt/C (40%) and Mo_2_TiC_2_T_*x*_−Pt_SA_. a–d) Reproduced with permission.^[^ ^15^ ^]^ Copyright 2018, Springer Nature. Comparison of e) polarization curves and f) calculated Δ*G*_H*_ for the Ti_3_C_2_T_*x*_, Ru_SA_−N−Ti_3_C_2_T_*x*_, Ru_NC_–Ti_3_C_2_T_*x*_, and N−Ti_3_C_2_T_*x*_ catalysts. e,f) Reproduced with permission.^[^ ^11^ ^]^ Copyright 2020, The Royal Society of Chemistry. g) Proposed reaction pathway on individual Pt of Pt_1_/Ti_3−*x*_C_2_T_*y*_. h) Comparison of calculated energy for Pt_1_/Ti_3−*x*_C_2_T_*y*_ and Pt nanoparticles (black line). g,h) Reproduced with permission.^[^ ^14^ ^]^ Copyright 2019, American Chemical Society.

Originating from the strong covalent interaction, the single Pt site anchored on MXene possesses a tiny |Δ*G*_H*_| of 0.08 eV (**Figure** **2**a), which is even smaller than that of commercial Pt catalysts (0.10 eV).^[^ ^15^ ^]^ Consequently, a low overpotential of 30 mV is required for Mo_2_TiC_2_T_*x*_−Pt_SA_ to achieve 10 mA cm^−2^, as shown in Figure 2b. Also, Mo_2_TiC_2_T_*x*_−Pt_SA_ exhibits Pt‐like kinetics with a small Tafel slope of 30 mV dec^−1^ (Figure 2c). It is noted that the Mo_2_TiC_2_T_*x*_−Pt_SA_ catalyst delivers a high mass activity, which is ≈40 times higher than the commercial Pt/C catalyst (40 wt%) based on the Pt loading, as shown in Figure 2d. In another work, single‐atom ruthenium (Ru_SA_) site supported on nitrogen (N)‐doped Ti_3_C_2_T_*x*_ MXene (N−Ti_3_C_2_T_*x*_) was constructed and used as an efficient HER catalyst at all pH media, which delivers high catalytic activity with overpotentials of 23, 37, and 81 mV at 10 mA cm^−2^ in 0.5 m H_2_SO_4_, 1.0 m KOH, and 1.0 m PBS conditions, respectively (Figure 2e).^[^ ^11^ ^]^ Density functional theory (DFT) calculations reveal that a tiny |Δ*G*_H*_| of 0.039 eV can be achieved via the strong coordinative interaction between Ru_SA_ and N‐doped Ti_3_C_2_T_*x*_, showing the promoted HER kinetics (Figure 2f). In addition, Wang and coworkers conducted the first‐principle calculations and predicted that the introduction of TM atoms (Fe, Co, Ni) on the surface of MXenes can weaken the strong binding of H*, reaching the optimal |Δ*G*_H*_|.^[^ ^29^ ^]^ This work provides theoretical guidance for exploration of single nonprecious metal atoms on MXenes.

SASs‐MXenes also exhibit optimized adsorption energy for other catalytic reactions. As discussed, CO_2_ is the main product generated from the combustion of fossil fuels, which leads to serve environmental issues, such as ocean acidification and global warming.^[^ ^30^ ^]^ Zhao et al. found that MXene vacancies‐confined single Pt atoms (Pt_1_/Ti_3−*x*_C_2_T_*y*_) are capable to efficiently convert CO_2_ to the value product through the CO_2_ hydrosilylation reaction.^[^ ^14^ ^]^ Their DFT calculations reveal that the atomically dispersed Pt atom can largely reduce the adsorption energy of reaction intermediates according to the Chalk−Harrod process (see Figure 2g,h), increasing the reaction kinetics. The earlier experimental and simulation results strongly suggested that the SASs‐MXenes could deliver the intrinsically improved kinetics for a range of reactions.

### 3.2 Cleaving the Strong Bonding of Gas molecules on SASs‐MXenes

Cleaving the strong bonding always requires high energy consumption, which is the key step for a range of chemical synthesis. Ammonia (NH_3_) is the precursor for the preparation of a series of important compounds, which can be obtained from the abundant nitrogen in atmosphere. However, the cleavage of N≡N requires huge energy input via the traditional Haber−Bosch synthesis process. The electrochemical nitrogen reduction is a cost‐effective and energy‐saving method to convert N_2_ to NH_3_. Luo et al. first studied the atomic sites on bare MXenes and found that exposed Ti‐edge atoms are more active for the electrocatalytic N_2_ fixation compared with the Ti atoms on the basal plane.^[^ ^31^ ^]^ Based on their DFT calculations, a smaller activation energy of 0.64 eV is required for the N_2_ absorbed on the Ti atoms on the edge plane compared with that (0.85 eV) on the basal plane, which matches well with previous computational predictions.^[^ ^32^ ^]^

**Figure 3:**
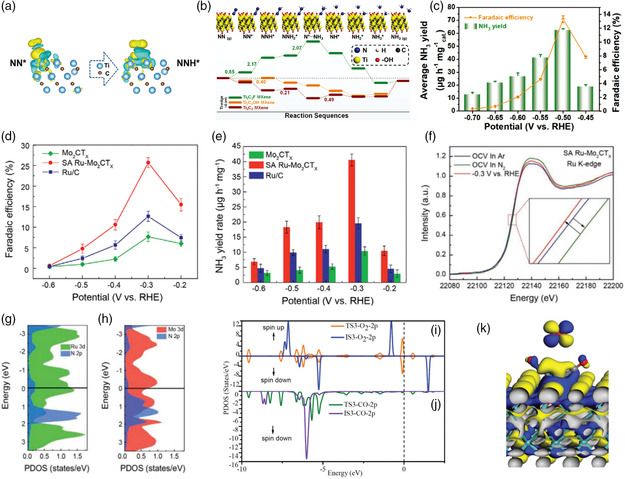
Activation of the gas molecules on SASs‐MXenes. a) The optimized charge density difference of the N_2_‐adsorbed configuration on the edge Ti of Ti_3_C_2_OH MXene surface. b) Reaction mechanism diagram and comparison of free‐energy schemes of NRR on the edge Ti of Ti_3_C_2_ MXene, Ti_3_C_2_F MXene, and Ti_3_C_2_OH MXene by distal pathway. c) Average NH_3_ yields and FEs for Ti_3_C_2_OH quantum dots/CP at different potentials. a–c) Reproduced with permission.^[^ ^33^ ^]^ Copyright 2020, Wiley‐VCH. Comparison of d) NH_3_ Faradaic efficiencies and e) the mass‐normalized NH_3_ yield rates at each potential for Mo_2_CT_*x*_, SA Ru−Mo_2_CT_*x*_, and commercial Ru/C. f) Operando XANES spectra at Ru *K*‐edge for SA Ru−Mo_2_CT_*x*_ under various conditions. The PDOS for the N atoms of *N_2_ directly bonded to g) Ru atoms and h) Mo atoms of SA Ru−Mo_2_CT_*x*_. d–h) Reproduced with permission.^[^ ^35^ ^]^ Copyright 2020, Wiley‐VCH. PDOS analysis of i) O_2_ and j) CO in TER‐IS3 and TER‐TS3 with the corresponding counterparts in the gas phases. k) A front view of the LUMO of a free O_2_ molecule and the HOMO of two CO coadsorbed on Pd/O_V_–Mo_2_CO_2_. The isosurface is set to be 0.01 e Å^−3^. i–k) Reproduced with permission.^[^ ^36^ ^]^ Copyright 2018, The Royal Society of Chemistry.

Jin et al. investigated the impact of surface terminals on the NRR activity of Ti‐edge atoms.^[^ ^33^ ^]^ As shown in **Figure** **3**a,b, they found that N_2_ can spontaneously adsorb on Ti_3_C_2_ and Ti_3_C_2_OH, whereas a higher energy barrier has to be overcome for N_2_ adsorption on Ti_3_C_2_F. As a result, hydroxyl‐rich Ti_3_C_2_T_*x*_ quantum dots deliver a Faradaic efficiency of 13.30% and NH_3_ yield of 62.94 μg h^−1^ mg^−1^ at −0.5 V versus reversible hydrogen electrode (RHE) (Figure 3c). The naturally existing SASs on bare MXenes show potential in NRR activity.^[^ ^34^ ^]^ To further improve the catalytic performance, the heteroatom sites are recommended to be introduced on MXenes.

Wei et al. reported single ruthenium atom‐modified MXene (SA Ru‐Mo_2_CT_*x*_), which can be used as the electrocatalyst for NRR. The SA Ru‐Mo_2_CT_*x*_ exhibits a Faradaic efficiency of 25.77% with a high ammonia yield rate of 40.57 μg h^−1^ mg^−1^ at −0.3 V versus RHE (Figure 3d,e), which outperforms those of pristine Mo_2_CT_*x*_ (faradaic efficiency [FE]: 7.73% and NH_3_ yield: 10.43 μg h^−1^ mg^−1^) and commercial Ru/C catalyst (FE: 12.71% an NH_3_ yield: 19.56 μg h^−1^ mg^−1^).^[^ ^35^ ^]^ Then they conducted a mechanism study via the operando X‐ray absorption spectroscopy (XAS). As shown in XANES spectra (Figure 3f), a higher valence state of Ru can be observed in N_2_‐saturataed electrolyte (+3.56) in comparison with the Ar_2_‐saturataed (+3.27), which could be caused by the formation of charge transfer from Ru atoms to the nitrogen species (N_2_^&−^), weakening the N ≡ N bond. As indicated by projected density of states (PDOS) (Figure 3g,h), a strong hybridization between adatom N_ads_ 2*p* orbitals and Ru 3*d* orbitals around the Fermi level implies a strong binding of *N_2_ species on Ru sites compared with Mo sites, which further suggests the important role single Ru sites play in cleaving N≡N bond, promoting the NRR kinetics.

SASs‐MXenes can be also potentially used to break the strong binding of other gas molecules. Cheng et al. predicted that the single Pd atom anchored on Mo_2_CO_2_ MXene can facilitate the O_2_ molecule activation and break the O—O bond, which can be used for CO oxidation.^[^ ^36^ ^]^ It is known that hydrogen is the main fuel for a proton exchange membrane fuel cell. The traditional method to produce hydrogen from the decomposition of alcohol may cause the contamination of CO in H_2_ fuel. When used in the fuel cell, the CO can poison the catalyst. It is demonstrated that the introduction of single Pd atoms on Mo_2_CO_2_ with oxygen defects can exhibit enhanced activity of CO oxidation, thus improving the efficiency of fuel cells. Furthermore, the analysis of PDOS shows that the partially occupied 2p* states of O_2_ in the TS3 structure weaken the O—O bond (Figure 3i,j). As shown in Figure 3k, the lowest unoccupied molecular orbital (LUMO) of the free O_2_ molecule matches well with the highest occupied molecular orbital (HOMO) of the two CO molecules adsorbed on the single Pd atom‐anchored Mo_2_CO_2_ monolayer, further demonstrating the electron transfer from CO to O_2_ 2p*orbital (LUMO), weakening the O—O bond. As indicated by the earlier results, the SASs‐MXenes show great capability to cleave the strong bond, which are promising catalysts for many sluggish reactions.

### 3.3 Decreasing the Energy Barrier of Potential‐Determining Step

**Figure 4:**
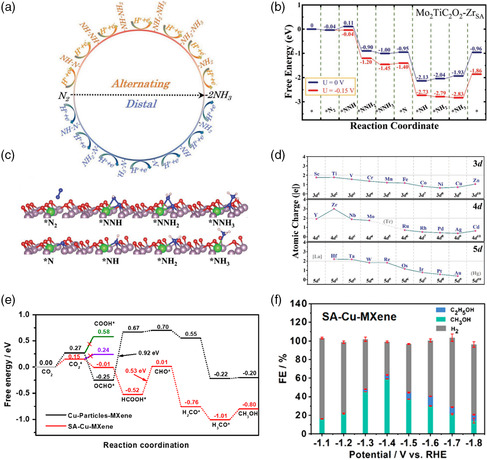
The illustration of reaction steps for NRR and DFT calculation results. a) Scheme of two possible NRR pathways on TM‐embedded Mo_2_TiC_2_O_2_. b) The free‐energy profiles at zero and limiting potentials through the distal path on Mo_2_TiC_2_O_2_−Zr_SA_. c) The configurations of key species during NRR. d) The atomic charges of the embedded TMs in the screened SACs. a–d) Reproduced with permission.^[^ ^39^ ^]^ Copyright 2019, Wiley‐VCH. e) Free energy diagram of CO_2_ to CH_3_OH on SA‐Cu‐MXene. f) FEs of SA‐Cu‐MXene at different potentials from −1.1 to −1.8 V. e,f) Reproduced with permission.^[^ ^24^ ^]^ Copyright 2021, American Chemical Society.

The potential‐determining step is the bottleneck for a range of electrocatalytic reactions.^[^ ^37^ ^]^ Decreasing the energy barrier of the potential‐determining step is key to achieving a highly efficient energy conversion accompanied with multiple‐electron transfer.^[^ ^38^ ^]^ A series of DFT calculations were conducted by Chen and coworkers to evaluate the electrocatalytic NRR performance for MXene (Mo_2_TiC_2_O_2_) vacancy‐confined TMs, including Zr, Mo, Hf, Ta, W, Re, and Os.^[^ ^39^ ^]^ As shown in **Figure** **4**a, there are two possible pathways for NRR, distal and alternation mechanisms. For the distal pathway, the distant N atom is firstly attacked, forming the NH_3_ molecule. Then the left N reacts with three proton–electron pairs to generate another NH_3_ molecule. For the alternating pathway, the hydrogenation processes alternately occur on both N atoms. Among all the vacancies‐confined TMs, Mo_2_TiC_2_O_2_−Zr_SA_ catalyst has the lowest energy barrier for potential‐determining step (*N_2_→step (which is predicted to be an efficient catalyst for NRR (Figure 4b,c). For a deeper understanding of the screened results, the Bader charge for a series of TMs on MXenes nanosheets should be studied. As shown in Figure 4d, the increase in the valence electrons leads to the decreased partial charge on single TMs. The polarized charged 5*d* TMs can efficiently activate the N_2_ molecule. Due to the unique electronic properties, single 5*d* TM atoms on MXenes are highly recommended to be investigated in multiple complex steps involving catalytic reactions.

In addition, the energy barriers of rate‐determining step (RDS) are the important parameters for catalysts, affecting their selectivity and efficiency in different reactions. As shown in Figure 4e, the SA‐Cu‐MXene with an unsaturated electronic structure (Cu^*δ*+^, 0 < *δ* < 2) offers a low RDS energy barrier (0.53 eV) for conversion of HCOOH* to absorbed CHO* compared with Cu particles‐MXene (0.92 eV for the conversion of OCHO* to HCOOH*).^[^ ^24^ ^]^ Consequently, the SA‐Cu‐MXene exhibits a high Faradaic efficiency of 59.1% to produce CH_3_OH via the electroreduction of CO_2_, in comparison with that of Cu particles‐MXene (14.6%) at the same potential (Figure 4f). The unsaturated atomic sites on MXenes are favorable to catalyze different multiple‐electron transfer reactions.

## 4 SASs‐MXenes for Energy Storage

The SASs‐MXenes are also broadly studied as high‐performance electrode material in various energy‐storage devices, which actively participate in the redox reactions on the electrode.^[^ ^40^ ^]^ In this section, we summarize the different roles SASs‐MXenes play in various storage devices, which include LMBs, Li−S batteries, and sodium/ZIBs. Overall, the introduction of SASs‐MXenes into the electrodes greatly improves the energy‐storage efficiency of these rechargeable batteries, leading to higher specific capacities and longer cycle life.

### 4.1 Inducing the Homogenous Li Nucleation on SASs‐MXenes

**Figure 5:**
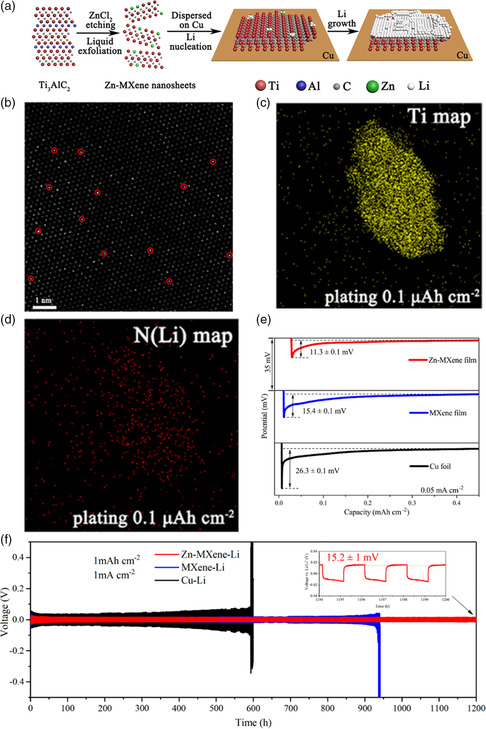
Inducing the homogenous Li nucleation on Zn‐MXene and its electrochemical performance. a) Scheme of synthesis procedures and Li nucleation process for Zn‐MXene. b) HAADF−STEM images of a Zn‐MXene nanosheet. Transmission electron microscopy mapping images for c) Ti and d) N (Li) on Zn‐MXene‐Li layers at the plating level of 0.1 μAh cm^−2^ after nitrogen labeling. e) Comparison of voltage−capacity curves of lithium plating on the resultant Zn‐MXene film, MXene film, and Cu foil measured at 50 μA cm^−2^. f) Cycle performances for symmetric cells composed of Zn‐MXene‐Li, MXene‐Li, and Cu−Li anodes at 1 mAh cm^−2^, respectively. a–f) Reproduced with permission.^[^ ^42^ ^]^ Copyright 2020, American Chemical Society.

The lithium metal anode has attracted great attention in next‐generation Li‐based batteries due to its high theoretical gravimetric capacity (3860 mAh g^−1^) and low potential (−3.04 V vs standard hydrogen electrode). However, the lithium ion tends to randomly grow on the metal anode, forming the dendrite. These lithium dendrites can pierce the separator, leading to poor cycle stability and even safety problems. Recently, Huang's group developed a green and HF‐free route to etch the “A” element in MAX phase using the Lewis acidic molten salt (ZnCl_2_, CuCl_2_, and FeCl_2_) synthesis approach.^[^ ^41^ ^]^ Our group used this method to fabricate the single zinc atoms on Ti_3_C_2_Cl_*x*_ (Zn‐MXene), as shown in **Figure** **5**a.^[^ ^42^ ^]^ The HAADF−STEM image (Figure 5b) shows that Zn atoms (bright dots) are precisely anchored at Ti sites, suggesting that the Zn atoms filled the cation vacancies. As shown in Figure 5c,d, the N (Li) element is homogeneously distributed on Zn‐MXene layers, which indicates that lithium tends to uniformly nucleate on Zn‐MXene layer. Then, the Zn‐MXenes film prepared by spray coating was used as the substrate for lithium plating. As shown in Figure 5e, a low nucleation potential can be achieved by Zn‐MXene film, compared with those of MXene film and Cu foil. Moreover, Zn‐MXene−Li anode shows long life up to 1200 h, and a small overpotential lower than 16 mV is maintained. Then, a full cell is fabricated by Zn‐MXene−Li anode and LiFePO_4_ cathode (Figure 5f), which possesses good cyclic stability and high Coulombic efficiency at 2 C (C = 170 mA g^−1^). Benefiting from the highly dispersed SASs on MXenes, the SASs‐MXenes can be also applied for sodium or zinc nucleation, suppressing the dendrite formation.

### 4.2 Accelerating the Rate‐Controlling Step of Polysulfides Conversion by SASs‐MXenes

**Figure 6:**
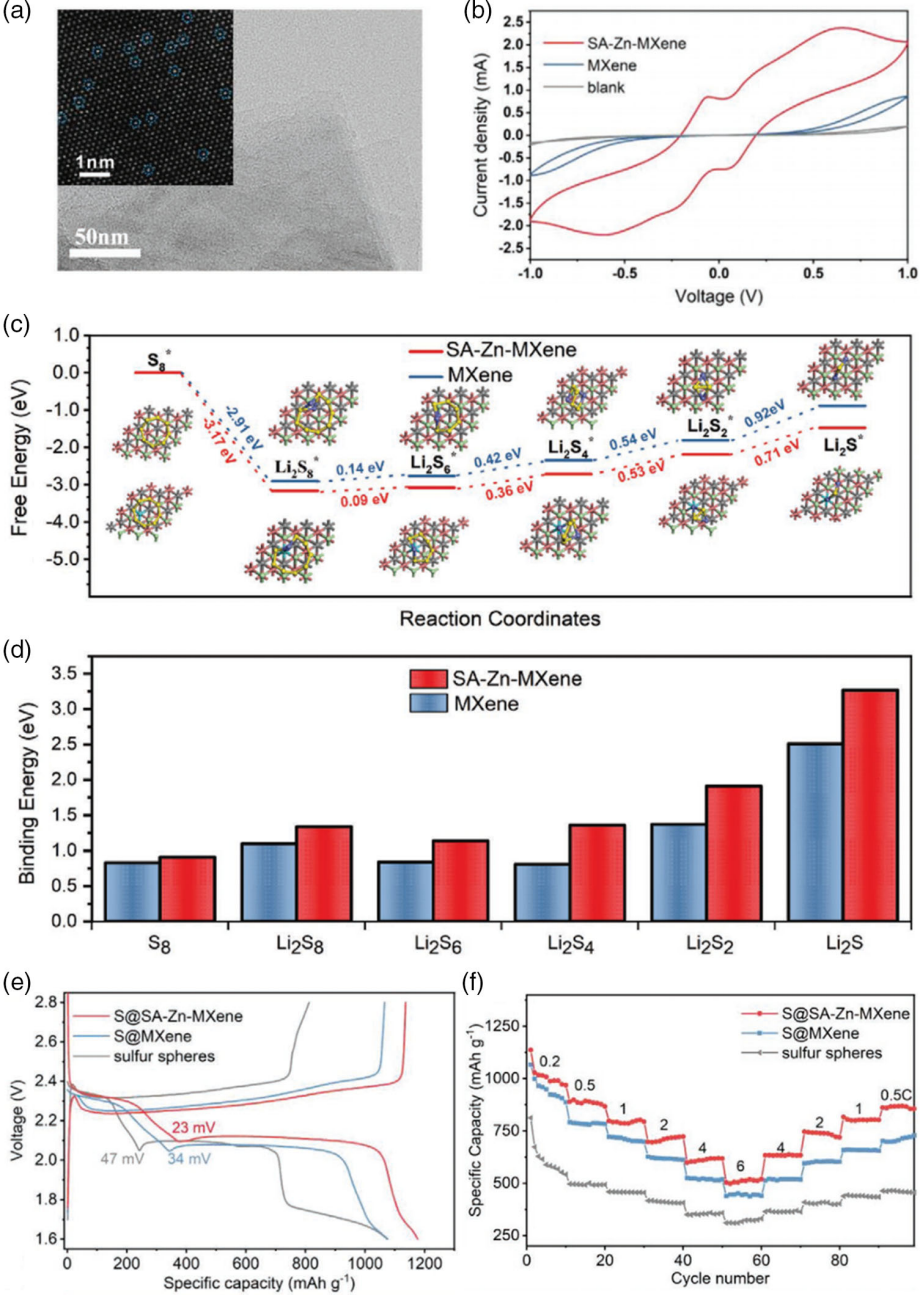
Accelerating polysulfides conversion by SA‐Zn‐MXene and achieving improved electrochemical performances. a) HAADF−STEM images of SA‐Zn‐MXene. b) Comparison of CVs for SA‐Zn‐MXene, MXene, and Al foil from −1.0 to 1.0 V at 3 mV s^−1^. c) Comparison of the Gibbs free‐energy profiles for lithium polysulfides on SA‐Zn‐MXene and MXene. d) Comparison of binding energies for lithium polysulfides with SA‐Zn‐MXene and lithium polysulfides with bare MXene. e) Discharge–charge profiles of S@SA‐Zn‐MXene, MXene, and sulfur spheres. f) Comparison of rate performances of S@SA‐Zn‐MXene, MXene, and sulfur spheres at different rates from 0.2 to 6 C. a–f) Reproduced with permission.^[^ ^43^ ^]^ Copyright 2020, Wiley‐VCH.

Li−S battery has long been considered a promising energy‐storage device due to high energy density (1675 mAh g^−1^) based on complex sulfur‐involved redox conversion reactions. However, the shuttle of highly soluble lithium polysulfides (LiPS) and sluggish conversion lead to the fast capacity decay and poor rate performance of sulfur cathode. To address the earlier issues, our group first introduced single‐atom zinc‐implanted MXene (SA‐Zn‐MXene) to sulfur cathode.^[^ ^43^ ^]^ The HAADF−STEM image (**Figure** **6**a) discloses the highly dispersed bright dots (Zn atoms) on the MXene nanosheet. The cyclic voltammetry (CV) measurement was carried out in a symmetric cell to evaluate the kinetics of polysulfide conversion reaction. As shown in CV curves (Figure 6b), much higher current densities can be achieved by SA‐Zn‐MXene compared with MXene in four peaks at −0.58, −0.07, 0.63, and 0.06 V, respectively, showing that the SA‐Zn‐MXene largely improves the kinetics of LiPS conversion. Further DFT calculations reveal that the SA‐Zn‐MXene largely reduces the energy barrier of the rate‐limiting step (from Li_2_S_2_ to Li_2_S), with a smaller free energy of 0.71 eV compared with that of the bare MXene (0.92 eV), as shown in Figure 6c,d. Then, the sulfur sphere was wrapped with SA‐Zn‐MXene (S@SA‐Zn‐MXene) to conduct the electrochemical test. As shown in Figure 6e, S@SA‐Zn‐MXene delivered a higher initial reversible capacity of 1136 mAh g^−1^ at 0.2 C compared with sulfur spheres wrapped with bare MXene (1065 mAh g^−1^) and pure sulfur spheres (812 mAh g^−1^). Moreover, the S@SA‐Zn‐MXene possesses a high reversible capacity of 517 mAh g^−1^ even at 6 C, in comparison with S@MXene (445 mAh g^−1^), presenting good rate capability as well (Figure 6f). In another study, Wang et al. reported a general strategy for atomically modifying the surface of MXene to achieve improved catalytic performance for polysulfides conversion.^[^ ^44^ ^]^ Ti_3_C_2_S_2_ with terminated S atom exhibits a small energy barrier of the decomposition process (Li_2_S→LiS + Li^+^ + e^−^), showing great potential as host for sulfur cathode. Originating from similar chemistry, the application for SASs‐MXenes can be extended to the Li−Se batteries to suppress polyselenides’ shuttle effect.^[^ ^45^ ^]^

### 4.3 Contribution of Pseudocapacitance by SASs‐MXenes

**Figure 7:**
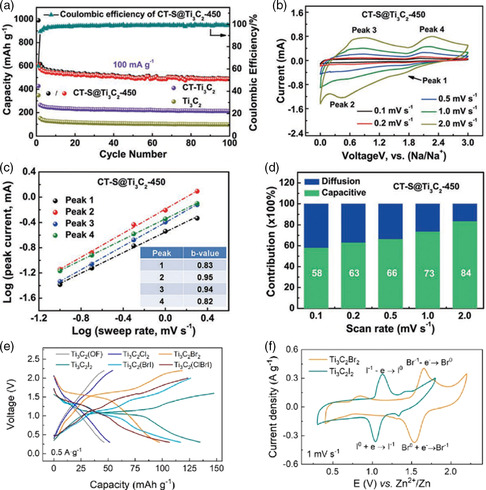
The electrochemical study of capacitive behaviors on SASs‐MXenes. a) Comparison of the cycling performance of Ti_3_C_2_, CT‐Ti_3_C_2_, and CT‐S@Ti_3_C_2_‐450 electrodes at 0.1 A g^−1^. b) CV curves of CT‐S@Ti_3_C_2_‐450 electrode at different scan rates. c) Logarithm peak current versus logarithm scan rate plots. d) Comparison of capacitive and diffusion‐controlled capacities contribution of CT‐S@Ti_3_C_2_‐450 electrode at different scan rates. a–d) Reproduced with permission.^[^ ^47^ ^]^ Copyright 2019, Wiley‐VCH. e) Typical galvanostatic charge‐discharge profiles curves of Ti_3_C_2_ MXenes with different terminals at the current density of 0.5 A g^−1^. f) CV curves of Ti_3_C_2_Br_2_ and Ti_3_C_2_I_2_ at 1 mV s^−1^. e,f) Reproduced with permission.^[^ ^48^ ^]^ Copyright 2021, American Chemical Society.

MXenes‐based materials have been widely used for ion storage, and the SASs on MXenes are closely related to the ion‐storage mechanism, thus affecting the energy storage‐capabilities.^[^ ^46^ ^]^ Luo et al. reported that the S atoms intercalated with Ti_3_C_2_ after annealing at 450 °C (CT‐S@Ti_3_C_2_‐450), which exhibit high capacity for sodium‐ion storage with 550 mAh g^−1^ at 0.1 A g^−1^ (**Figure** **7**a).^[^ ^47^ ^]^ Then, the electrochemical kinetics study was conducted (Figure 7b–d), revealing that the surface‐controlled capacitive contribution dominates the sodium storage. Moreover, a high capacitive contribution of 84% can be achieved at a scan rate of 2 mV s^−1^, determining the high rate capability for sodium‐ion storage.

Recently, Huang and coworkers synthesized Ti_3_C_2_ MXene terminated with −Br (Ti_2_C_2_Br_2_) and −I (Ti_3_C_2_I_2_), showing the capacities of 97.6 and 135 mAh g^−1^ for zinc‐ion storage with distinct discharging platforms (Figure 7e).^[^ ^48^ ^]^ As shown in Figure 7f, the characteristic redox peaks observed in both Ti_3_C_2_I_2_ (1.05/1.15 V) and Ti_3_C_2_Br_2_ (1.55/1.65 V) represent the reversible conversion of I^−^/I^0^ and Br^−^/Br^0^, respectively. The terminated atomic sites are redox active, which can be directly used as active sites for ion storage. This work points out a new concept for taking the advantages of the modified terminated atom sites for efficient energy storage.

## 5 Conclusion and Perspectives

SASs‐MXenes have exhibited great significance in both fundamental research and practical applications. We compared the different synthetic strategies for anchoring the single atoms on MXenes by taking advantages of the defective and terminated spots. The loading of single atoms on MXenes may vary from different preparation methods, which would impact the physical and chemical properties (**Table** **1**). With more utilizations of advanced characterization techniques and computational simulations in the study of SASs‐MXenes, a deep understating from the mechanism perspective is given. In this Review, we mainly disclosed the key roles SASs‐MXenes played in improving the kinetics of various electrochemical reactions.

**Table 1:** Overview of the reported SASs‐MXenes for energy conversion and storage

Materials	Reactions	Mass loading of single atoms	Performances	Ref.
Mo_2_CT_*x*_:Co	HER	0.04 wt%	η_10_ = 180 mV	[23]
Mo_2_TiC_2_T_*x*_−Pt_SA_	HER	1.2 wt%	*η*_10_ = 30 mV	[15]
Ru_SA_−N−Ti_3_C_2_T_*x*_	HER	1.1 wt%	*η*_10_ = 23 mV	[11]
Ru_SA_−N−S−Ti_3_C_2_T_*x*_	HER	1.2 wt%	*η*_10_ = 76 mV	[20]
Pt_1_/ Ti_3−*x*_C_2_T_*y*_	Functionalization of CO_2_	0.2 wt%	Amide selectivity: ≈100%	[14]
Ru_1_/Ti_3−*x*_C_2_T_*y*_	N/A	0.08 wt%	N/A	[14]
Ir_1_/Ti_3−*x*_C_2_T_*y*_	N/A	0.19 wt%	N/A	[14]
Rh_1_/Ti_3−*x*_C_2_T_*y*_	N/A	0.05 wt%	N/A	[14]
Pd_1_/Ti_3−*x*_C_2_T_*y*_	N/A	0.04 wt%	N/A	[14]
SA‐Ru−Mo_2_CT_*X*_	NRR	1.41 wt%	NH_3_ yield: 40.57 μg h^−1^ mg^−1^ at −0.3 V versus RHE	[35]
SA‐Zn‐MXene	Polysulfides conversion	1.5 wt%	640 mAh g^−1^ at 6 C	[43]
SA‐Cu‐MXene	CRR	1.0 wt%	FE(CH_3_OH) = 59.1% at −1.4 V versus RHE	[24]

Although great progress has been made for the development of SASs‐MXenes, it is still challenging for controllable and scalable synthesis of single atoms on MXenes with high loading and uniform dispersion to meet the high demands for practical applications. For instance, the high‐temperature treatment may not only cause the aggregation of single atoms but also the stacking of MXene nanosheets, which greatly reduce the number of active sites.^[^ ^49^ ^]^ In addition, the randomly distributed cation vacancies formed during etching of MAX phase under harsh conditions may lead to the inhomogeneous distribution of single atoms trapped on these vacancies. The poor oxidation stability of MXenes also has to be considered during the synthesis processes.^[^ ^50^ ^]^ To address these issues, the following aspects for the future development of highly active SASs‐MXenes are rationally proposed.

### 5.1 Synergistic Effect between Single Atoms and Different MXenes Substrates

Previous studies have proved that the SASs‐MXenes can exhibit unusual physical and chemical properties, which originate from the strong interactions of single atoms and MXenes substrate. When using the cation vacancies to capture the single atoms, the appropriate MXenes substrate has to be selected to accommodate single atoms with different radii. As the stability of SASs is closely related to their coordinated atoms, the MXenes substrate with proper terminated groups is preferred for certain single atoms.^[^ ^51^ ^]^ Therefore, choosing the right MXenes substrates may lead to further improved catalytic performance and stability of SASs. To date, more than 30 MXenes have been reported.^[^ ^52^ ^]^ The diversified phase compositions provide a variety of local electronic environments for SASs, which inspire great research efforts to further explore the potential of SASs‐MXenes for other reactions.

### 5.2 Single‐Atom Array on MXenes

**Figure 8:**
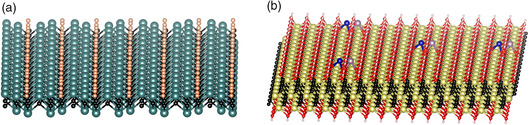
The proposed atomic models for future SASs‐MXenes developments. a) Single‐atom array on MXenes. b) Dual‐atom pair on MXenes. The yellow, cayan, black, and red spheres represent Ti, Mo, C, and O; blue, pink, orange spheres represent TMs.

The precise control of SASs on MXenes is a fascinating scheme, which bridges the gap between the atomic structural features and performance. Rosen et al. reported 2D Mo_1.33_C with ordered divacancies by selectively etching the Al and Sc atoms in (Mo_2/3_Sc_1/3_)_2_AlC.^[^ ^53^ ^]^ These ordered divacancies are perfect sites for trapping single atoms. As shown in **Figure** **8**a, the single‐atom array with high loading and an ordered arrangement can be achieved. More interestingly, the single‐atom array may exhibit unusual properties different from both the bulk and isolated forms, showing great research significance. Moreover, a single‐atom array with ordered in‐plane structure may possess high stability than single atoms individually dispersed on MXenes, which is promising for practical applications.

### 5.3 Dual‐Atom Pair on MXenes

Recently, dual‐atom pair has attracted much attention to regulate the intrinsic catalytic properties. The different atomic centers can perform synergistically, optimizing the adsorption of the reaction intermediates. So far, the dual‐atom pair has been anchored on carbon‐based materials, showing greatly enhanced performance.^[^ ^54^ ^]^ We proposed dual‐atom pair on MXenes (Figure 8b), which may help to further accelerate the kinetics in multiple‐electron‐transfer reactions. As different active centers control different reaction steps, dual‐atom pair may work well together, improving the catalytic performance. We believe that the dual‐atom pair on MXenes can be foreseen soon.

## Conflict of Interest

The authors declare no conflict of interest.
